# *Plantago asiatica* L. Seed Extract Improves Lipid Accumulation and Hyperglycemia in High-Fat Diet-Induced Obese Mice

**DOI:** 10.3390/ijms18071393

**Published:** 2017-06-30

**Authors:** Qiming Yang, Meng Qi, Renchao Tong, Dandan Wang, Lili Ding, Zeyun Li, Cheng Huang, Zhengtao Wang, Li Yang

**Affiliations:** 1 The Ministry of Education (MOE) Key Laboratory for Standardization of Chinese Medicines and the State Administration of Traditional Chinese Medicine (SATCM) Key Laboratory for New Resources and Quality Evaluation of Chinese Medicines, Institute of Chinese Materia Medica, Shanghai University of Traditional Chinese Medicine, Shanghai 201203, China; nicholasyang_tcm@yahoo.com (Q.Y.); uranusm@163.com (M.Q.); tongqiyun932@163.com (R.T.); xzmqdd@126.com (D.W.); nail8219@126.com (L.D.); lizeyun2006@163.com (Z.L.); 2 School of Pharmacy, Shanghai University of Traditional Chinese Medicine, Shanghai 201203, China; chuang.shutcm@gmail.com

**Keywords:** *Plantago asiatica* L. seed, high-fat diet, mice, obesity, lipid accumulation, hyperglycemia, metabolic disorder, peroxisome proliferator activated receptor signaling

## Abstract

Obesity and its common association with type 2 diabetes, dyslipidemia, and cardiovascular diseases are worldwide epidemics. Currently, to prevent or treat obesity and associated metabolic disorders, herbal dietary supplements or medicines have attracted more and more attention owing to their relative effectiveness with fewer significant side effects. We investigate the therapeutic effects and underlying mechanisms of *Plantago asiatica* L. seed extract (PSE) on obesity and associated metabolic disorders in high-fat (HF) diet-induced mice. Our results displayed that PSE did not modify food intake or body weight but decreased abdominal white adipose tissue ratio, white/brown adipocyte size, serum total cholesterol, triglyceride (TG), low density lipoprotein cholesterol, free fatty acid, and hepatic TG concentrations when compared with the HF group. The levels of fasting blood glucose and glucose tolerance were improved in the PSE group when compared with the HF group. Furthermore, PSE upregulated mRNA expressions of peroxisome proliferator activated receptors (PPARs) and target genes related to fatty acid metabolism and energy expenditure in liver and adipose tissue of obese mice when compared with the HF group. PSE treatment effectively improved lipid and glucose metabolism in HF diet-induced obese mice. These effects might be attributed to the upregulation of PPAR signaling

## 1. Introduction

Obesity, which is caused by a combination of excessive food energy intake and lack of physical exercise, normally leads to increased health risks of type 2 diabetes, dyslipidemia, atherosclerosis, hypertension, cardiovascular diseases, or certain types of cancer [1,2,3]. Reduction of energy-dense food consumption and exercise are the main treatments for obesity [4]. In recent years, the use of dietary supplements has become an effective strategy to prevent or to treat obesity and associated metabolic disorders [5,6]. Traditional herbal medicines contain various active compounds, such as polysaccharides, flavonoids, terpenes, alkaloids, and iridoids, and many of them exhibit valid anti-obesity effects [7,8,9].

*Plantago asiatica* L. (Chinese plantain) is a common herbal medicine belonging to the genus *Plantago* and is native to East Asia. This herb is traditionally used to treat liver disease, stomach problems, and urinary system inflammation [10,11,12]. *P. asiatica* L. leaves are consumed as a vegetable or tea in Japan and northeastern China. *P. asiatica* L. seed, which is recorded as Plantaginis Semen in Chinese pharmacopoeia, is well known for its diuretic, antipyretic, antitussive, antiphlogistic, and defecation-facilitating properties [13,14]. Recent reports have demonstrated that many *Plantago* species exerted therapeutic effects on obesity, type 2 diabetes, and lipid disorders [15,16,17]. In previous studies, a methanol extract of *P. asiatica* L. showed glycation inhibitory activity and blocked the development of diabetic complications [18]. The polysaccharides from *P. asiatica* L. seeds lowered the apparent lipid absorption in mice by modulating colon microbiota [19,20]. The essential oils in *P. asiatica* L. exerted hypolipidemic effects on C57BL/6 mice [21]. These reports suggested that *P. asiatica* L. seeds might potentially participate in the amelioration of glucose and lipid metabolism and obesity. 

## 2. Results

### 2.1. Chemical Constituents of the Plantago asiatica L. Seed Extract (PSE)

We determined the chemical composition of PSE by ultra-performance liquid chromatography–mass spectrometry (UPLC-MS). In the chromatographic profile of PSE (Figure S1), plantagoguanidinic acid A and caffeic acid were detected in positive ionization scan mode, whereas geniposidic acid, plantamajoside, acteoside, and isoacteoside were detected in negative mode. The peaks of these compounds were identified by comparison with the UPLC retention time and MS spectra of the standard compounds. The relative amounts of the main compounds of PSE, namely acteoside, geniposidic acid, and plantagoguanidinic acid A, were, respectively, 27.7, 42.3 and 6.83 mg/g PSE, which were equivalent to 4.42, 6.77, and 1.09 mg/g in crude *P. asiatica* L. seed.

### 2.2. The Effects of PSE on the Body Weight, Food Intake and Lipid Accumulation of High-Fat (HF) Diet-Induced Obese C57BL/6 Mice

**Figure 1:**
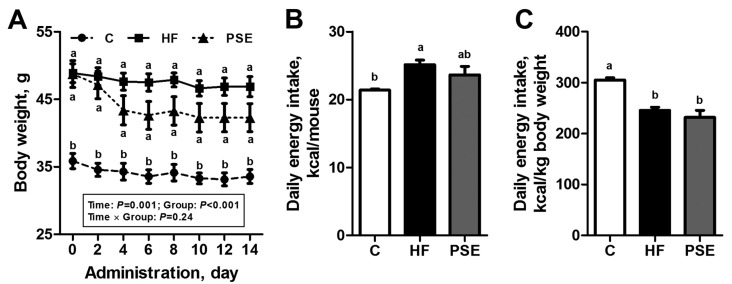
Body weight changes (**A**); mean energy intake per mouse (**B**); and per kg body weight (**C**) of 16-week HF diet-induced obese C57BL/6 mice after being treated with PSE for two weeks. Data are presented by means ± SEMs; *n* = 8 per group (**A**); and *n* = 10 per group (**B**,**C**). Body weight among groups was analyzed by two-way repeated-measures ANOVA with between (groups) and within (days) subject factors, and data on each day were compared by one-way multivariate ANOVA with Bonferroni post hoc test. Multiple comparisons of energy intake were analyzed by one-way ANOVA with Bonferroni post hoc test. Labeled means (**B**,**C**); or means on one day (**A**) without a common letter significantly differ at *p* < 0.05 (a > b). C, control; HF, high-fat; PSE, *Plantago asiatica* L. seed extract.

After 16 weeks of a HF diet, C57BL/6 mice increased their body weight by 39.4% compared with the Chow diet group (*p* < 0.001). The body weights of PSE treatment for two weeks decreased by about 10% compared with HF group, although no significant difference was observed (*p* = 0.16) (Figure 1A). Slightly lower amount of absolute food intake in the PSE group was detected compared with the HF group, and the difference was not significant (*p* = 0.66); the difference in the food intake corrected by body weight was also not significant (*p* = 0.94) (Figure 1B,C).

**Figure 2:**
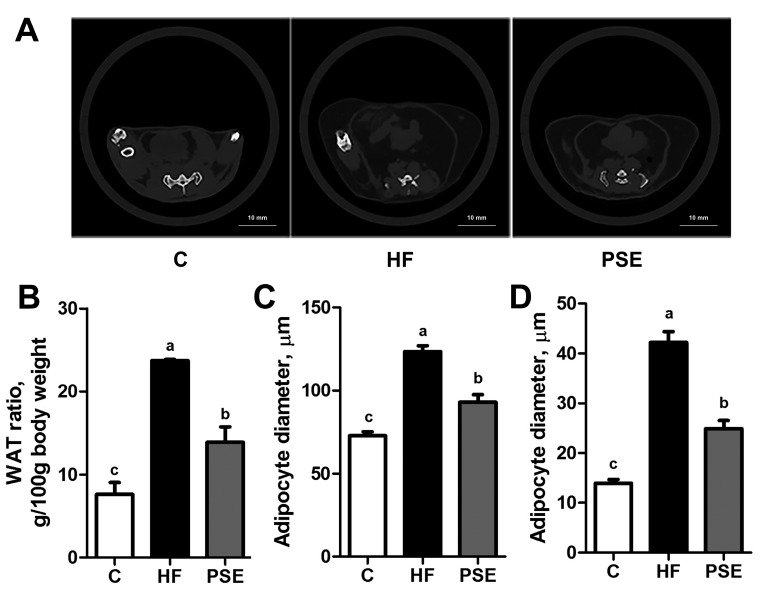
Computed tomography scans at 8 mm above the top of the iliac bone (**A**); abdominal WAT ratios (**B**); diameters of white adipocytes (**C**); and brown adipocytes (**D**) of 16-week HF diet-induced obese C57BL/6 mice after treatment with PSE for two weeks. Data are presented as means ± SEMs; *n* = 4 per group (**B**); and *n* = 8 per group (**C**,**D**). One-way ANOVA with Bonferroni post hoc test was used for statistical analysis. Labeled means without a common letter significantly differ at *p* < 0.05 (a > b > c). WAT, white adipose tissue.

**Figure 3:**
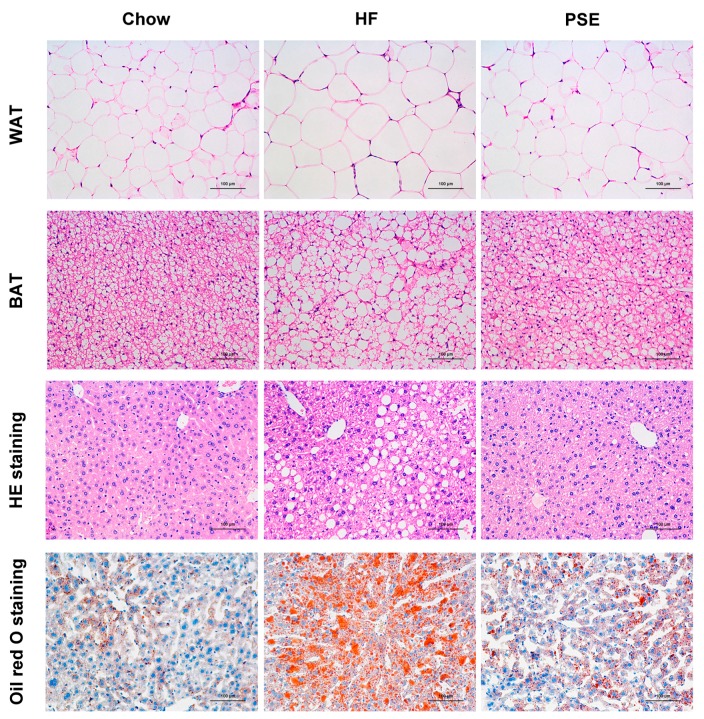
Histological analysis of liver, WAT, and BAT after two weeks of PSE treatment in 16-week HF diet-induced obese C57BL/6 mice. Liver tissue sections were stained with oil red O or H&E (×200) to observe liver lipid content. WAT and BAT were stained with H&E (×200) to observe adipocyte size. BAT, brown adipose tissue; H&E, hematoxylin and eosin. Scale bar: 100 µm.

Remarkable lipid accumulation in abdomen was displayed with greater white adipose tissue (WAT) ratio, i.e., WAT weight (g) per body weight (100 g), in HF group than in Chow diet group (*p* < 0.001). However, PSE treatment reduced lipid accumulation by 41.5% compared with HF diet group (*p* < 0.001) (Figure 2A,B). In the pathological examination with hematoxylin and eosin (H&E) and oil red O staining (Figure 3), PSE treatment alleviated the macrovesicular steatosis induced by HF diet. After PSE treatment for two weeks, the diameters of white adipocytes declined from 124 ± 3.52 μm to 93.0 ± 4.44 μm (*p* < 0.001), and brown adipocytes from 42.2 ± 2.21 μm to 24.9 ± 1.61 μm (*p* < 0.001) (Figure 2C,D).

**Figure 4:**
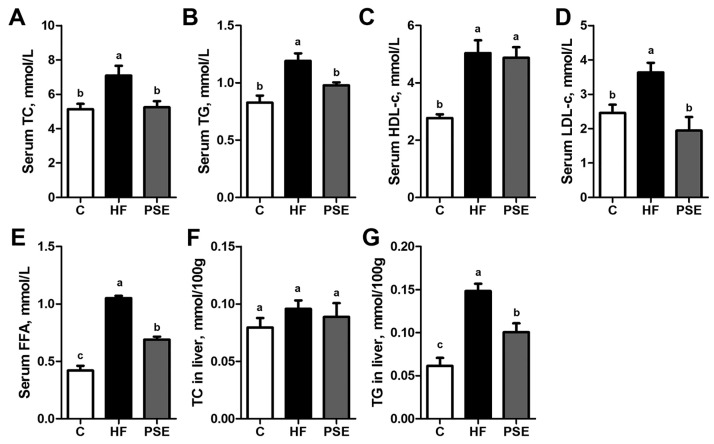
Serum TC (**A**); TG (**B**); HDL-c (**C**); LDL-c (**D**); FFA (**E**); liver TC (**F**); and liver TG (**G**) concentrations in 16-week HF diet-induced obese C57BL/6 mice after PSE treatment for two weeks. Data are presented as means ± SEMs; *n* = 8 per group. Statistical analyses were performed by one-way ANOVA with Bonferroni post hoc test. Labeled means without a common letter significantly differ at *p* < 0.05 (a > b > c). FFA, free fatty acid; HDL-c, high density lipoprotein cholesterol; LDL-c, low density lipoprotein cholesterol; TC, total cholesterol; TG, triglyceride.

As shown in Figure 4, the serum total cholesterol (TC) (*p* = 0.016), triglyceride (TG) (*p* < 0.001), high density lipoprotein cholesterol (HDL-c) (*p* < 0.001), low density lipoprotein cholesterol (LDL-c) (*p* = 0.045), free fatty acid (FFA) (*p* < 0.001), and liver TG (*p* < 0.001) concentrations in the HF group were significantly increased compared with those in the Chow diet group. PSE administration notably decreased the serum TC, TG, LDL-c, FFA, and liver TG concentrations to 73.8% (*p* = 0.030), 82.1% (*p* = 0.040), 53.4% (*p* = 0.003), 65.7% (*p* < 0.001), and 67.8% (*p* = 0.007) of HF, respectively, but did not affect serum HDL-c concentration. Liver TC concentrations did not alter among the three groups.

### 2.3. The Effects of PSE on Glucose Homeostasis of HF Diet-Induced Obese C57BL/6 Mice

**Figure 5:**
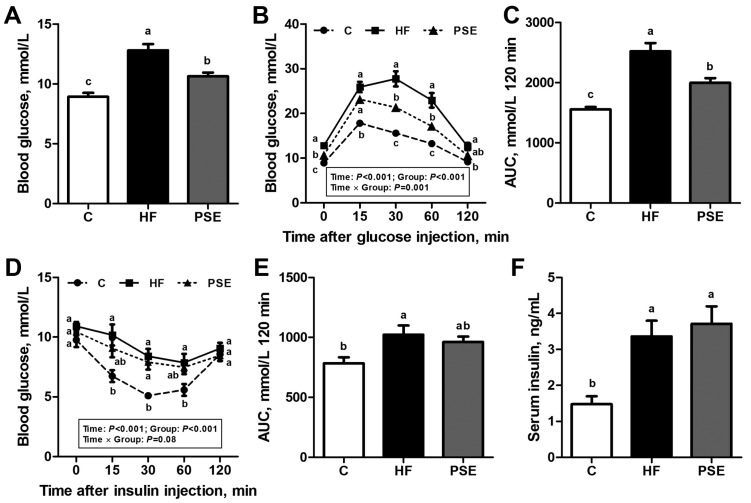
Effects of two weeks PSE treatment on glucose metabolic disorders in 16-week HF diet-induced obese C57BL/6 mice. (**A**) Blood glucose concentrations after 8 h of fasting at 0 min; and (**B**) GTT. After measuring the baseline fasting blood glucose levels, the mice were intraperitoneally injected with 1 g glucose/kg body weight and the subsequent blood glucose concentrations at 15, 30, 60, and 120 min were measured; (**C**) the AUC from the GTT in (**B**); and (**D**) ITT. Non-fasting blood glucose levels were measured at 0 min, and the blood glucose concentrations at 15, 30, 60, and 120 min were tested after intraperitoneal injection of 0.75 U insulin/kg body weight; (**E**) The AUC from the ITT in (**D**); and (**F**) serum insulin level after 8 h of fasting. Data are presented as means ± SEMs; *n* = 8 per group. GTT and ITT were performed by two-way repeated-measures ANOVA with between (groups) and within (minutes) subject factors, and data in each time point were compared by one-way multivariate ANOVA with Bonferroni post hoc test. Other multiple comparison analyses were performed by one-way ANOVA with Bonferroni post hoc test. Labeled means (**A**,**C**,**E**,**F**); and means at a time point (**B**,**D**) without a common letter significantly differ at *p* < 0.05 (a > b > c). AUC, area under the curve; GTT, glucose tolerance test; ITT, insulin tolerance test.

The fasting blood glucose of mice in the HF group was elevated to 12.8 ± 0.511 mmol/L and 43.6% greater than in the Chow diet group (*p* < 0.001). The HF group also exhibited impaired glucose and insulin tolerance. PSE administration significantly lowered the HF-induced increase in fasting blood glucose concentrations (*p* = 0.002) (Figure 5A). After glucose injections in glucose tolerance test (GTT), blood glucose concentrations in the PSE group were significantly decreased at 30 min (*p* = 0.001) and 60 min (*p* = 0.002) compared with those in HF group (Figure 5B). However, the PSE group did not exhibit significant changes in blood glucose concentrations after insulin injections (Figure 5D). The areas under the curve (AUCs) of GTTs and insulin tolerance tests (ITTs) from 0 to 120 min in Chow diet, HF, and PSE groups showed the similar results (Figure 5C,E). Meanwhile, the fasting serum insulin concentrations were significantly increased in the HF diet-induced obese mice (*p* = 0.004), whereas not decreased by PSE administration (*p* = 1.00). HOMA-indexes for evaluating insulin resistance (HOMA-IR), sensitivity (HOMA-IS), and beta cell function (HOMA-β) were also calculated [22]. No significant differences of HOMA-indexes were displayed between HF and PSE groups (HOMA-IR, *p* = 1.00; HOMA-IS, *p* = 1.00; HOMA-β, *p* = 0.25) as shown in Figure S2. The above results suggested that PSE could reduce HF-induced hyperglycemia but not hyperinsulinemia.

### 2.4. PSE Regulates Lipid and Glucose Metabolism Related Gene Expressions

**Figure 6:**
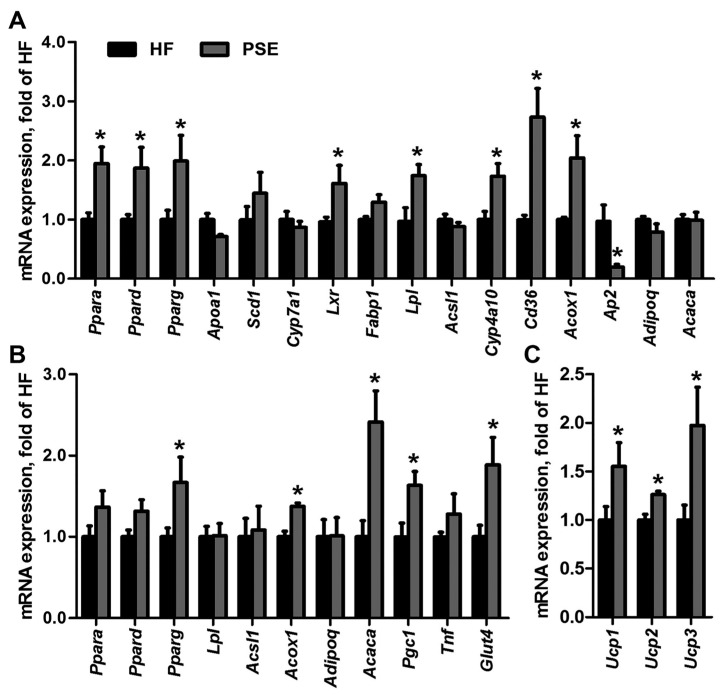
Alterations of mRNA expression levels of PPARs and their target genes in: liver (**A**); WAT (**B**); and BAT (**C**) of 16-week HF diet-induced obese C57BL/6 mice after treatment with PSE for two weeks. For each group, equal amounts of total RNA from the tissues of 6 mice were subjected to qRT-PCR as described in the Methods. β-Actin was used as the internal control. The data are presented as means ± SEMs from three independent experiments. Statistical analyses were performed by Student’s t test. * *p* < 0.05 versus HF. Acaca, acetyl-Coenzyme A carboxylase α; Acox1, acyl-Coenzyme A oxidase 1; Acsl1, acyl-CoA synthetase long-chain family member 1; Adipoq, adiponectin; Ap2, fatty acid binding protein 4; Apoa1, apolipoprotein A-I; Cd36, cluster of differentiation 36; Cyp, cytochrome P450; Fabp1, fatty acid binding protein 1; Glut4, glucose transporter type 4; Lpl, lipoprotein lipase; Lxr, liver X receptor α; Pgc1, PPAR gamma coactivator 1 α; Ppar, peroxisome proliferator activated receptor; Scd1, stearoyl-Coenzyme A desaturase 1; Tnf, tumor necrosis factor α; Ucp, uncoupling protein.

Differential gene expressions between HF and PSE groups were determined by microarray analysis and were considered significant when *p* value was <0.05 and the fold change was >2. The gene matrixes of HF and PSE groups (*n* = 3) were categorized into respective distinct clusters by hierarchical classification (Figure S3A). A total of 285 genes in PSE group were significantly different compared with those in HF group, among which 123 and 162 genes were upregulated and downregulated, respectively. Functional pathway analysis was applied in KEGG and Biocarta database, and the pathways were designated as significant when enrichment test *p* value was <0.05. PSE-altered genes were involved in 57 significant pathways (24 upregulated and 33 downregulated pathways). Among these pathways, three upregulated pathways (peroxisome proliferator activated receptor (PPAR) signaling pathway, fatty acid metabolism, and type II diabetes mellitus), and 10 downregulated pathways (steroid biosynthesis, mitogen-activated protein kinase (MAPK) signaling pathway, terpenoid backbone biosynthesis, retinol metabolism, sterol regulatory element-binding protein (SREBP) control of lipid synthesis, fatty acid biosynthesis, regulation and function of carbohydrate-responsive element-binding protein (ChREBP) in liver, synthesis and degradation of ketone bodies, primary bile acid biosynthesis, and vascular smooth muscle contraction) were associated with liver function, cholesterol synthesis and degradation, fatty acid metabolism, glucose homeostasis, and blood pressure regulation (Figure S3B). PPARs play essential roles in energy metabolism, fatty acid/cholesterol synthesis and transport, and have been recognized as drug targets for metabolic diseases. PPARs have three subtypes, namely PPARα, PPARβ/δ, and PPARγ, which show different distributions and specific functions. Therefore, a reporter assay was performed to analyze the effects of PSE on the transcription activities of PPARα, PPARδ, and PPARγ. PSE alone at 200 μg/mL increased PPARα and PPARγ transactivities (*p* = 0.046, *p* = 0.039), but were not as strong as when the agonists alone were used (*p* < 0.001, *p* < 0.001). PSE at 200 μg/mL combined with agonists significantly activated PPARα and PPARγ transcriptions (*p* < 0.001, *p* = 0.002) compared with the agonists alone, which indicated that PSE may have a strong synergistic effect with the agonists (Figure S4). However, PSE displayed no effect on PPARδ transactivities. The result indicated that PSE may contain agonists of PPARα and PPARγ. Furthermore, we evaluated the mRNA expression levels of PPARs and related target genes in liver, WAT, and brown adipose tissue (BAT) of mice in the HF and PSE groups. The mRNA expression levels of Ppara (*p* = 0.006), Ppard (*p* = 0.027), Pparg (*p* = 0.045), and their target genes, including liver X receptor α (Lxr) (*p* = 0.043), lipoprotein lipase (Lpl) (*p* = 0.018), cluster of differentiation 36 (Cd36) (*p* = 0.006), cytochrome P450 4a10 (Cyp4a10) (*p* = 0.010), and acyl-Coenzyme A oxidase 1 (Acox1) (*p* = 0.021), in liver tissues in the PSE group were significantly greater than those in the HF group. However, the hepatic expression of fatty acid binding protein 4 (Ap2) was suppressed (*p* = 0.013) (Figure 6A). Moreover, PSE administration increased the mRNA expression levels of Pparg (*p* = 0.049), Acox1 (*p* < 0.001), acetyl-Coenzyme A carboxylase α (Acaca) (*p* = 0.004), PPAR gamma coactivator 1 α (Pgc1) (*p* = 0.017), and glucose transporter 4 (Glut4) (*p* = 0.031) in WAT of HF diet-induced obese mice (Figure 6B). We also analyzed the gene expressions of thermogenic markers uncoupling protein 1 (Ucp1), Ucp2, and Ucp3 in BAT of HF and PSE groups. As shown in Figure 6C, the expression levels of Ucp1 (*p* = 0.046), Ucp2 (*p* = 0.001), and Ucp3 (*p* = 0.041) in HF diet-induced obese mice were greatly upregulated after PSE treatment. These results suggested that PSE likely improved lipid and glucose metabolism by enhancing PPAR signaling.

## 3. Discussion

*Plantago asiatica* L. seed is commonly used as food and medicine in many Asian countries and has diuretic, antioxidant, immunoregulatory, and hepaprotective properties [13,14,23]. In recent years, *P. asiatica* L. seeds or PSE have showed biological effects on bacterial infection, inflammation, hypertension, liver impairment, hypercholesterolemia, and advanced glycation end-product formation [24,25,26]. Previous studies have revealed that *P. asiatica* L. seeds contain high amounts of fatty acids, flavones, polysaccharides, iridoids, phenylpropanoid glycosides, and guanidine derivatives [27,28,29]. UPLC-MS analysis of PSE in our study showed that the extract mainly contained geniposidic acid, acteoside, isoacteoside, and plantagoguanidinic acid A. Previous studies suggested that geniposidic acid and other iridoid glycosides can protect against liver disease, hypertension, diabetes, and obesity-induced metabolic function disorders [30,31,32,33]. Iridoid glycosides exert prevention and improvement on abnormal glucolipid metabolism via multiple approaches, such as reducing inflammatory reactions caused by oxidative stress and the formation of advanced glycation endproducts, inhibiting α-glucosidase activity and adipocyte differentiation, as well as activating LXR and PPAR [32,34,35,36]. Acteoside and isoacteoside exist widely in herbal medicines and exhibit multiple beneficial effects against metabolic disorders based on hepatoprotection and inhibition of angiotensin-converting enzyme and α-glucosidase activities [34,37,38]. Previous studies report the herb extracts rich in phenylpropanoid glycosides have anti-obesity effects on diet-induced mice, and the possible mechanisms were demonstrated as stimulating leptin expression and cholesterol catabolism, suppressing fatty acid synthesis, and accelerating fatty acid β-oxidation [39,40]. Plantagoguanidinic acid A is a newly found guanidine derivative from *P. asiatica* L. seeds, and improves glucose metabolism in alloxan-induced diabetic mice [29,41,42]. Other guanidine derivatives were also reported to have potential anti-inflammatory and anti-hyperglycemic effects in in vitro pharmacological studies through inhibition of cyclooxygenase-2 activity and suppression of hepatic gluconeogenesis [43,44]. Thus, the effects of PSE on lipid and glucose metabolic disorders may have been through the co-action of iridoids, phenylpropanoid glycosides, and guanidine derivatives.

C57BL/6 mice with a long-term intake of HF diet could develop metabolic syndrome, which includes obesity, fatty liver, dyslipidemia, impaired fasting blood glucose, and insulin resistance [45,46]. In this study, PSE treatment for two weeks considerably limited the lipid accumulation in adipose tissues and liver of obese mouse. Glucose metabolism, which was impaired by HF diet, improved after PSE therapy. PSE lowered the body weight gain induced by HF diet, but the change was not significant. The absolute food intake per mouse in the PSE group showed a slight and insignificant difference with that in the HF group, and this difference may have potentially influenced weight loss. However, PSE was inadequate in alleviating abnormal serum HDL-c and insulin concentrations caused by the HF diet. Insulin resistance induced by HF was not ameliorated after PSE treatment, indicating that the improvement glucose homeostasis induced by PSE was not through the improvement of insulin resistance. Therefore, the exact reason should be explored further. Nevertheless, the results demonstrated that PSE exerted therapeutic effect against HF diet-induced hepatic steatosis, hyperlipidemia, and hyperglycemia.

To further investigate the mechanism underlying the amelioration of metabolic disorders by PSE, genome expression microarray analysis was presented to determine the significantly different pathways that were involved in lipid and glucose metabolism between HF and PSE groups. The results showed that pathways of PPAR signaling, fatty acid metabolism, SREBP control of lipid synthesis, and fatty acid biosynthesis were modulated by PSE treatment. Among these pathways, PPAR signaling pathway was closely related to regulation of carbohydrate and lipid levels, and was further investigated in our experiment. PPARs are a group of nuclear receptors expressed in liver, adipose tissues, and muscles, and play essential roles in the regulation of lipogenesis, adipogenesis, and glucose homeostasis [47]. PPARs, when activated, are molecular targets for the treatment of dyslipidemia and type 2 diabetes [48]. Previous studies showed that many herbal products exert effects on metabolic disorders via PPAR activation [49]. In the present study, we revealed that PSE activates PPARα and PPARγ transcriptions through transfection and reporter gene assays, thereby indicating that PSE may contain natural PPARα and PPARγ agonists. We then investigated the mRNA expression levels of PPARs and related target genes in tissues of mice in the HF and PSE groups. PPARs mediate lipid and glucose homeostasis by regulating the expression of target genes related to lipid metabolism, adipocyte differentiation, and adaptive thermogenesis [50]. In this study, PSE significantly increased the mRNA expressions of PPARs and some target genes in liver, WAT, and BAT. Among these target genes, Lpl and Cd36 are involved in TG hydrolysis and play important role in fatty acid transport and lipid accumulation [51]. Cyp4a10, Acox1, and Acaca are related to the synthesis and oxidation of fatty acids, and the increasing expression of which could prevent dietary steatohepatitis [50]. Ap2 gene modulates adipocyte differentiation and lipid accumulation, and blocking its protein may be a potent therapeutic strategy for metabolic disorders [49,50]. Ucp1, Ucp2, and Ucp3 expressed in BAT could generate heat after being activated by fatty acids and could play roles in nonshivering thermogenesis, diabetes, and obesity [52]. The mRNA expression levels of Ucp1, Ucp2, and Ucp3 in BAT of mice in the PSE group were significantly increased. Thus, energy expenditure might have been increased by PSE, thereby resulting in body weight loss. Moreover, our results showed that the mRNA expression levels of Pgc1 and Glut4 in WAT were increased by PSE. PGC1 is a co-activator of PPARγ and plays a vital role in energy metabolism, lipogenesis, and lipoprotein in liver [53]. GLUT4, which is mainly expressed in adipose tissues and striated muscles, is an insulin-regulated glucose transporter and could stimulate glucose intake by the cells [54]. Therefore, we considered that PSE ameliorated lipid and glucose metabolic disorders mainly through the upregulation of mRNA expression of PPARs and some target genes related to fatty acid metabolism and energy expenditure in PPAR signaling.

## 4. Materials and Methods

### 4.1. Extract Preparation and UPLC-MS Analysis

The extract from dried *Plantago asiatica* L. seeds was prepared and analyzed by UPLC-MS as described in the Supplementary Materials and Methods.

### 4.2. Experimental Animals and Diet

Male C57BL/6 mice (6 weeks old, SPF) were purchased from Slac Laboratory (Shanghai, China). After 1 week of adaptation, mice were fed with a control diet containing 10% kcal from fat (Research Diets, New Brunswick, NJ, USA; D12450B) or a HF diet containing 60% kcal from fat (Research Diets, New Brunswick, NJ, USA; D12492) for 18 weeks. At Week 16, mice fed with the HF diet were induced obesity [45,55] and randomized to 2 groups (PSE and HF group, *n* = 8/group) with treatment of PSE at 1.44 g/(kg·day) or equal volume of water by gavage for the remaining two weeks. The group of mice fed with the control diet was used as a control group (C group, *n* = 8). PSE dose was determined based on our previous study of its anti-diabetes and anti-obesity effects. Each group of mice was housed in two plastic cages. The individual body weights of mice and food intakes were recorded every other day. The mean food intake per mouse and per body weight was calculated. The mice were maintained under constant temperature (22 ± 2 °C) and relative humidity (60–65%) with a 12 h light-dark cycle (dark at 1900). Water and food were provided ad libitum. All experiments were performed in compliance with the guidelines for the care and use of laboratory animals as approved by the Animal Ethics Committee of Shanghai University of Tradition Chinese Medicine on 22 October 2014 with the approval number SZY 2014031.

### 4.3. Computed Tomography Scan Analysis

The abdominal adiposity of mice was examined using Latheta LCT-200 system (Hitachi-Aloka, Tokyo, Japan) according to the manufacturer’s protocol. Computed tomography scan was performed at 2 mm intervals on abdominal region to determine WAT ratio.

### 4.4. Intraperitoneal Glucose and Insulin Tolerance Tests

In accordance to the previous papers [56,57], GTTs were performed after 8 h of fasting. For ITTs, the random blood glucose levels (0 min) of all mice were determined without fasting. The AUCs of the GTTs and ITTs from each group were calculated [55]. Serum insulin levels after 8-h fasting were determined by ELISA kit according to the instructions of the manufacturer (Mercodia AB, Uppsala, Sweden).

### 4.5. Serum and Hepatic Lipid Concentrations

At the end of experimental period, we collected blood samples from the hearts of mice after an 8-hour fasting. The liver tissues were collected rapidly after sacrificing the animals, frozen in liquid nitrogen, and stored at −80 °C for the following experiments. The hepatic TG and TC were extracted and measured by following the reported methods [56,58]. TC, TG, HDL-c, LDL-c, and FFA concentrations in serum and liver samples were measured by using the commercial analysis kits obtained from Nanjing Jiancheng Institute of Biotechnology (Nanjing, China). 

### 4.6. Histological Analysis

Fresh liver, WAT, and BAT samples were fixed in 10% formaldehyde and then paraffin-embedded for H&E staining. Another portion of liver tissue was fixed in 4% paraformaldehyde and embedded in OCT (Sakura Finetek, Torrance, CA, USA) for oil red O (Sigma-Aldrich, St. Louis, MO, USA) staining. The sections were examined under light microscope (×200) by using the Olympus image analysis software system (Olympus America, Center Valley, PA, USA).

### 4.7. Microarray Hybridization

RNA purification from the livers of HF and PSE mice (*n* = 3), and genome microarray hybridization were described in the Supplementary Materials and Methods. The data were processed under SBC Analysis System [59] from Shanghai Biotechnology Corporation, Ltd. (Shanghai, China) for differential gene expression, hierarchical clustering, and pathway analysis.

### 4.8. Transfection and Reporter Gene Assays

The transfection mixture was prepared [60] and added to HEK293T cells (obtained from ATCC) for 24-hour incubation, and then replaced to fresh media containing PPARα, PPARδ, and PPARγ agonists, WY14643, GW0742, and rosiglitazone (10 μM; Sigma-Aldrich, St. Louis, MO, USA), or PSE (50, 100, and 200 μg/mL) to incubate for another 24 h. The luciferase activities were determined according to the manuscript of Dual-Luciferase Reporter Assay System (Promega, Madison, WI, USA). The renilla luciferase activity was assayed to normalize transfection efficiencies. All transfection experiments were performed in triplicate and repeated at least triplicate independently.

### 4.9. qRT-PCR Analysis

Total RNA in liver, WAT, and BAT were extracted using spin column (Shanghai Generay Biotech Co., Ltd., Shanghai, China) and reversed into cDNA (TaKaRa, Kusatsu, Japan) according to the manufacturer’s protocol. The gene expression levels were determined by qRT-PCR using Applied Biosystems ViiA 7 Real-Time PCR system (Life Technologies, Singapore). The primers used in the experiments are listed in Table S1. The cDNA in a 10 μL reaction volume was denatured at 95 °C for 30 s followed by 40 cycles of PCR (95 °C, 5 s, 60 °C, 30 s). β-Actin was used as an internal control to normalize the expression levels of genes.

### 4.10. Statistical Analysis

All data were statistically analyzed by using SPSS 16.0 software (SPSS Inc., Chicago, IL, USA) and were presented as means ± SEMs. Body weight, GTT, and ITT curves were performed by two-way repeated-measures ANOVA with between (different groups) and within (different time points) subject factors, and data in each time point were compared by one-way multivariate ANOVA (Bonferroni post hoc test). Measurement of PPAR transcription factor signaling was not repeated over time, and thus, the data were analyzed by two-way ANOVA. Other multiple comparisons among three or more groups were analyzed by one-way ANOVA with Bonferroni post hoc test. Means were significantly different at *p* < 0.05 if they were labeled without a common lowercase letter (a > b > c). Besides, Student’s t test was used to analyze the differences of gene expression levels between HF and PSE groups, and means labeled with * were considered statistically significant at *p* < 0.05. 

## 5. Conclusions

In conclusion, our study demonstrated that PSE exerted therapeutic effects against obesity, hyperlipidemia, hepatic lipid accumulation, hyperglycemia, and glucose intolerance in HF diet-induced obese mice. These effects may involve activation of multimolecular targets in PPAR signaling pathway. These findings suggested that PSE can be used as a potential dietary supplement to treat or prevent obesity and relative symptoms. Further investigations should be made to define the composition of PSE and screen the PPAR agonists for therapy of metabolic disorders. 

## Supplementary Materials

Supplementary materials can be found at www.mdpi.com/1422-0067/18/7/1393/s1.

## Abbreviations

Acaca	acetyl-Coenzyme A carboxylase α
Acox1	acyl-Coenzyme A oxidase 1
Acsl1	acyl-CoA synthetase long-chain family member 1
Adipoq	adiponectin
Ap2	fatty acid binding protein 4
Apoa1	apolipoprotein A-I
AUC	area under the curve
BAT	brown adipose tissue
C	control
Cd36	cluster of differentiation 36
ChREBP	carbohydrate-responsive element-binding protein
Cyp	cytochrome P450
Fabp1	fatty acid binding protein 1
FFA	free fatty acid
Glut4	glucose transporter type 4
GTT	glucose tolerance test
HDL-c	high density lipoprotein cholesterol
H&E	hematoxylin and eosin
HF	high-fat
HOMA-IS	HOMA-insulin sensitivity
HOMA-β	HOMA-beta cell function
ITT	insulin tolerance test
LDL-c	low density lipoprotein cholesterol
Lpl	lipoprotein lipase
Lxr	liver X receptor α
MAPK	mitogen-activated protein kinase
Pgc1	PPAR gamma coactivator 1 α;
PPAR	peroxisome proliferator activated receptor
PSE	*Plantago asiatica* L. seed extract
Scd1	stearoyl-Coenzyme A desaturase 1
SREBP	sterol regulatory element-binding protein
TC	total cholesterol
TG	triglyceride
Tnf	tumor necrosis factor α
Ucp	uncoupling protein
UPLC-MS	ultra-performance liquid chromatography-mass spectrometry
WAT	white adipose tissue
