# Supplementary material for: Plantago asiatica L. Seed Extract Improves Lipid Accumulation and Hyperglycemia in High-Fat Diet-Induced Obese Mice
Source: Int J Mol Sci. 2017 Jun 30;18(7):1393. doi: 10.3390/ijms18071393 (PMC5535886; doi:10.3390/ijms18071393)
Supplement: Supplementary file 1 [file ijms-18-01393-s001.pdf]

Plantago Asiatica L. Seed Extract Improves Lipid Accumulation and Hyperglycemia in High-Fat Diet-Induced Obese Mice

Qiming Yang, Meng Qi, Renchao Tong, Dandan Wang, Lili Ding, Zeyun Li, Cheng Huang, Zhengtao Wang * and Li Yang *

Supplemental Materials and Methods

Plant material and extraction. Dried seeds of *Plantago asiatica* L. were purchased from Kangqiao Pharmaceutical Co., Ltd. (Shanghai, China) and the identification was confirmed by Lihong Wu, Institute of Chinese Materia Medica, Shanghai University of Traditional Chinese Medicine, China. Appropriate amount of powdered seeds was weighed and immerged into 10 times its volume of 60% alcohol overnight, followed by 3 reflux extractions for 2 h each. Filtrates were combined, concentrated under reduced pressure, and freeze-dried over 24 h to provide *Plantago asiatica* L. seed extract (PSE). The extraction yield from the crud drug was 15.9% (g/g). The extract was stored at −20 °C and dissolved with distilled water before administration to the mice.

Ultra-performance liquid chromatography-mass spectrometry (UPLC-MS) analysis of the extract. The UPLC-MS analysis of the extract was performed by a Waters Acquity UPLC system (Waters Corp., Milford, MA, USA) combined with a Micromass ZQ quadruple mass spectrometer (Waters Corp., Manchester, UK) equipped with electrospray ionization. Waters Acquity UPLC BEH C18 column (1.7 μm, 100 × 2.1 mm i.d.) protected by Acquity UPLC BEH C18 VanGuard pre-column (1.7 μm, 5 × 2.1 mm i.d.) were used for the chromatographic separation with the column temperature at 45 °C. The mobile phase was 0.1% formic acid (A)-acetonitrile (B) at a flow rate of 0.3 mL/min in gradient elution as follows: 0–4 min, 5–15% B; 4–7 min, 15–25% B; 7–9 min, 90% B; 9–10 min, 5% B. In the MS analysis, full scan in both positive and negative ionization mode was used with the following parameters: capillary voltage, 2.5 kV; sample cone, 35 V; extraction cone, 4.0 V; source temperature, 120 °C; desolvation temperature, 400 °C. Nitrogen, used as desolvation and cone gas, was set as 600 and 50 L/h, respectively. Instrumental control and data collection were carried out by MassLynx V4.1 software (Waters Corp., Milford, MA, USA).

Microarray hybridization. Frozen livers were homogenized and total RNA was isolated using TRIzol reagent (Invitrogen, Carlsbad, CA, USA). The RNA purity and concentration were confirmed by Nanodrop ND-1000 UV-VIS spectrophotometer (Thermo Scientific, Waltham, MA, USA), and the assessment of RNA integrity was identified with Agilent 2100 BioAnalyzer (Agilent Technologies, Santa Clara, CA, USA). The RNA samples were purified with RNeasy Mini Kit (Qiagen, Hilden, Germany), followed by cDNA synthesis, cRNA fluorescent labeling and purification according to the protocol (Agilent Technologies). Probes were hybridized with Agilent Whole Mouse Genome Oligo Microarray (4 × 44 K) chips (Agilent Technologies) for 17 h. After wash, the slides were scanned by Agilent microarray scanner (Agilent Technologies).

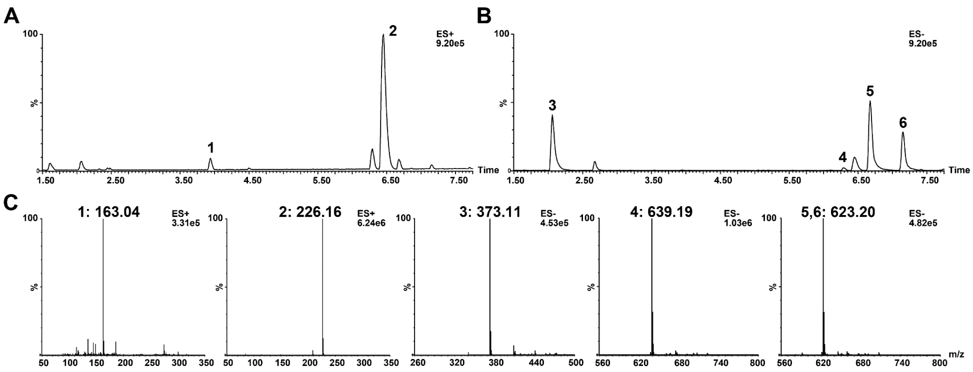

Figure S1. The total ion chromatograms of PSE and mass spectrums of standards by UPLC-MS technique. (**A**) Chromatogram in positive ionization mode; (**B**) chromatogram in negative ionization mode; (**C**) mass spectrums of standards: (1) caffeic acid; (2) plantagoguanidinic acid A; (3) geniposidic acid; (4) plantamajoside; (5) acteoside; (6) isoacteoside. PSE, *Plantago asiatica* L. seed extract; UPLC-MS, ultra-performance liquid chromatography-mass spectrometry.

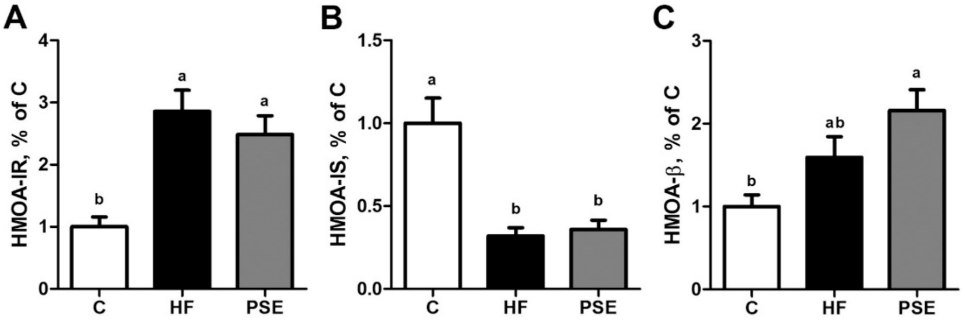

Figure S2. HOMA-IR (**A**), HOMA-IS (**B**) and HOMA-β (**C**) of HF diet-induced obese C57BL/6 mice after treated with PSE for 2 weeks. Data are presented as means ± SEMs; $n = 8$ per group. Statistical analyses were performed by one-way ANOVA with Bonferroni *post-hoc* test. Labeled means without a common letter significantly differ at $p < 0.05$ (a > b). C, control; HF, high-fat; HOMA-IS, HOMA-insulin sensitivity; HOMA-β, HOMA-β cell function; PSE, *Plantago asiatica* L. seed extract.

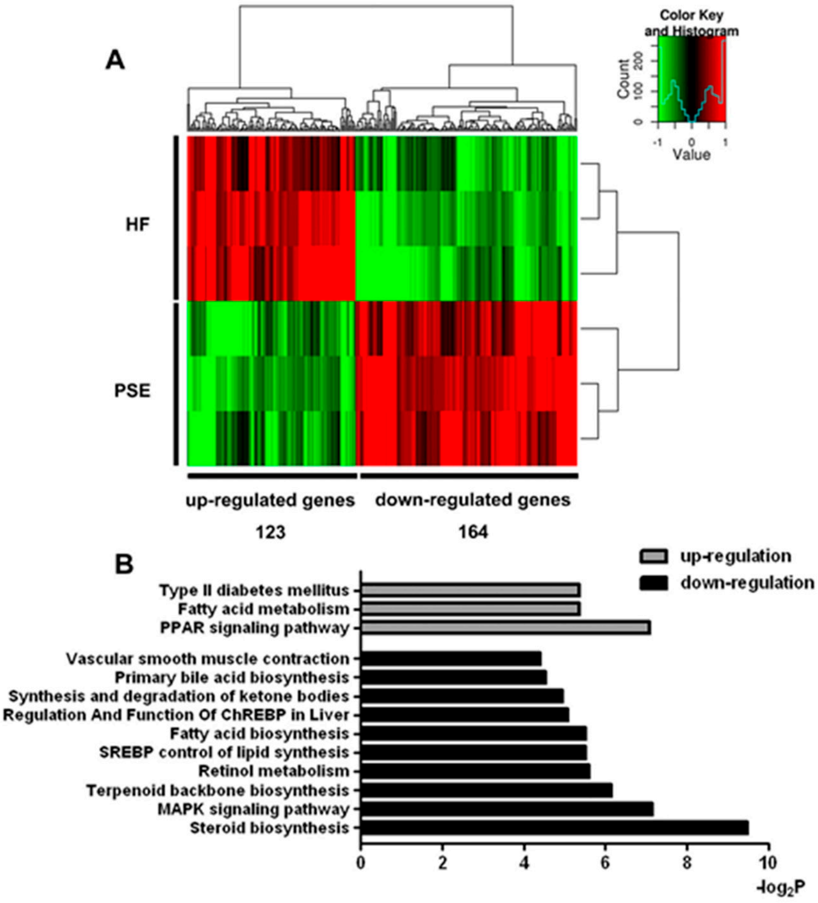

Figure S3. Differentially hepatic gene expression and pathway analysis between HF and PSE groups (*n* = 3). (**A**) Heat map of the differentially expressed genes ($p < 0.05$, fold change > 2). Red color indicated overexpressed genes, while green color indicated the opposite. The color scale bar is shown; (**B**) significant pathways for up- and down-regulated genes ($p < 0.05$). -Log2P meant the base 2 logarithm of the P value. ChREBP, carbohydrate-responsive element-binding protein; MAPK, mitogen-activated protein kinase; PPAR, peroxisome proliferator activated receptor; SREBP, sterol regulatory element-binding protein.

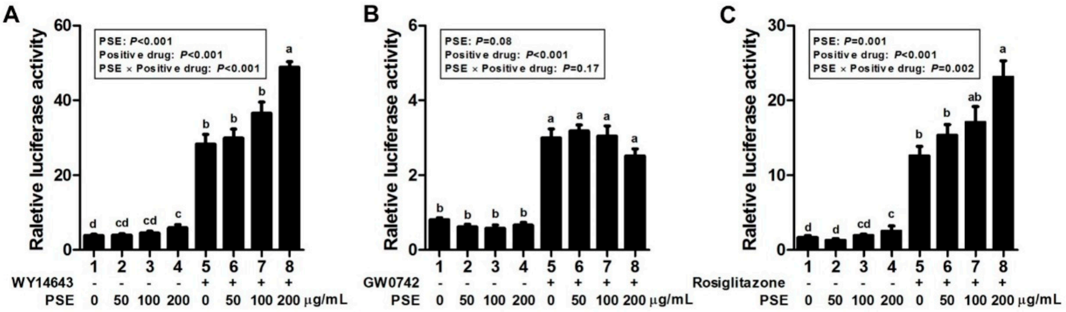

Figure S4. Activation of PSE on the transcription activities of PPARα (**A**), PPARδ (**B**) and PPARγ (**C**) though in vitro reporter gene assays. Renilla luciferase was used as a transfection efficiency control. The data are presented as means ± SEMs from three independent experiments; *n* = 6 per group. Statistical analyses were performed with two-way ANOVA. Labeled means without a common letter significantly differ at $p < 0.05$ (a > b > c > d).

Table S1. Sequences of the primers used in qRT-PCR.

Gene	5′-Forward primer-3′	5′-Reverse primer-3′
β-Actin	GGCTGTATTCCCCTCCATCG	CCAGTTGGTAACAATGCCATGT
Ppara [1]	AGAGCCCCATCTGTCCTCTC	ACTGGTAGTCTGCAAAACCAAA
Ppard	TCCATCGTCAACAAAGACGGG	ACTTGGGCTCAATGATGTCAC
Pparg	TCGCTGATGCACTGCCTATG	GAGAGGTCCACAGAGCTGATT
Acaca	ATGGGCGGAATGGTCTCTTTC	TGGGGACCTTGTCTTCATCAT
Acox1	AGAACCCATTTGCACACCTTG	AGCGTCCGTATCTTGAGTCCT
Acsl1	TGCCAGAGCTGATTGACATTC	GGCATACCAGAAGGTGGTGAG
Adipoq	TGTTCCTCTTAATCCTGCCCA	CCAACCTGCACAAGTTCCCTT
Ap2	TTTTTCAGCTATGGACCGTCAC	GAAGTCGGCATTAGGGGTGTG
Apoa1	GGCACGTATGGCAGCAAGAT	CCAAGGAGGAGGATTCAAACTG
Cd36	TTTGGAGTGGTAGTAAAAAGGGC	TGACATCAGGGACTCAGAGTAG
Cyp4a10	TTCCCTGATGGACGCTCTTTA	GCAAACCTGGAAGGGTCAAAC
Cyp7a1	GGGATTGCTGTGGTAGTGAGC	GGTATGGAATCAACCCGTTGTC
Fabp1	GGAATTGGGAGTAGGAAGAGCC	TGGACTTGAACCAAGGAGTCAT
Glut4	GTGACTGGAACACTGGTCCTA	CCAGCCACGTTGCATTGTAG
Lpl	GGGAGTTTGGCTCCAGAGTTT	TGTGTCTTCAGGGGTCCTTAG
Lxr	CTCAATGCCTGATGTTTCTCCT	TCCAACCCTATCCCTAAAGCAA
Pgc1	TATGGAGTGACATAGAGTGTGCT	CCACTTCAATCCACCCAGAAAG
Scd1	TTCTTGCGATACACTCTGGTGC	CGGGATTGAATGTTCTTGTCGT
Tnf	CCCTCACACTCAGATCATCTTCT	GCTACGACGTGGGCTACAG
Ucp1	AGGCTTCCAGTACCATTAGGT	CTGAGTGAGGCAAAGCTGATTT
Ucp2	ATGGTTGGTTTCAAGGCCACA	CGGTATCCAGAGGGAAAGTGAT
Ucp3	CTGCACCGCCAGATGAGTTT	ATCATGGCTTGAAATCGGACC

[1] Acaca, acetyl-Coenzyme A carboxylase α; Acox1, acyl-Coenzyme A oxidase 1; Acsl1, acyl-CoA synthetase long-chain family member 1; Adipoq, adiponectin; Ap2, fatty acid binding protein 4; Apoa1, apolipoprotein A-I; Cd36, cluster of differentiation 36; Cyp, cytochrome P450; Fabp1, fatty acid binding protein 1; Glut4, glucose transporter type 4; Lpl, lipoprotein lipase; Lxr, liver X receptor α; Pgc1, PPAR gamma coactivator 1 α; Scd1, stearoyl-Coenzyme A desaturase 1; Tnf, tumor necrosis factor α; Ucp, uncoupling protein.
